# Therapy Approaches for Stargardt Disease

**DOI:** 10.3390/biom11081179

**Published:** 2021-08-09

**Authors:** Elena Piotter, Michelle E McClements, Robert E MacLaren

**Affiliations:** 1 Nuffield Laboratory of Ophthalmology, Department of Clinical Neurosciences, University of Oxford, Oxford OX3 9DU, UK; elena.piotter@ndcn.ox.ac.uk (E.P.); enquiries@eye.ox.ac.uk (M.E.M.); 2 Oxford University Hospitals NHS Foundation Trust NIHR Biomedical Research Centre, Oxford OX3 9DU, UK

**Keywords:** Stargardt disease, gene therapy, inherited retinal disease, CRISPR

## Abstract

Despite being the most prevalent cause of inherited blindness in children, Stargardt disease is yet to achieve the same clinical trial success as has been achieved for other inherited retinal diseases. With an early age of onset and continual progression of disease over the life course of an individual, Stargardt disease appears to lend itself to therapeutic intervention. However, the aetiology provides issues not encountered with the likes of choroideremia and X-linked retinitis pigmentosa and this has led to a spectrum of treatment strategies that approach the problem from different aspects. These include therapeutics ranging from small molecules and anti-sense oligonucleotides to viral gene supplementation and cell replacement. The advancing development of CRISPR-based molecular tools is also likely to contribute to future therapies by way of genome editing. In this we review, we consider the most recent pre-clinical and clinical trial data relating to the different strategies being applied to the problem of generating a treatment for the large cohort of Stargardt disease patients worldwide.

## 1. Introduction

Stargardt disease is an inherited macular degeneration that typically presents in the first two decades of life [1]. The prevalence is in the region of 1 in 8–10,000 individuals. A recent British Ophthalmological Surveillance Unit (BOSU) study reported an annual incidence of 0.127 per 100,000; however, this postal-based questionnaire has a notoriously low reply rate from clinicians, due to the administrative burden resulting from identifying positive cases [2]. Stargardt disease patients typically make up a large proportion of patient cohorts within genetic clinics of eye disease [3,4]. The age of onset and rate of progression vary greatly, with most experiencing symptoms in their teens or earlier, and virtually all becoming severely visually impaired or legally blind by their 4th to 7th decade of life [1]. Given the relatively slow disease progression, there is a reasonable window for treatment intervention and an opportunity to improve or delay further degeneration throughout the lifetime of an individual. A diagnosis of Stargardt disease includes varying phenotypic and genotypic forms, of which STGD1 is the most common. This an autosomal recessive condition caused by mutations in the gene ABCA4, which encodes the ATP binding cassette protein family member 4. In rare cases, Stargardt-like disease arises that often falls under the umbrella term of Stargardt disease. Stargardt-like disease is caused by autosomal dominant mutations in ELOVL4 (STGD3) or PROM1 (STGD4), which encode elongation of very-long-chain fatty acids and prominin 1, respectively. Note that the term STGD2 was discontinued in 2005, when it was discovered to be the same gene as STGD3 (https://www.ncbi.nlm.nih.gov/gene/6784, accessed on 30 June 2021).

### 1.1. STGD1—ABCA4

**Figure 1:**
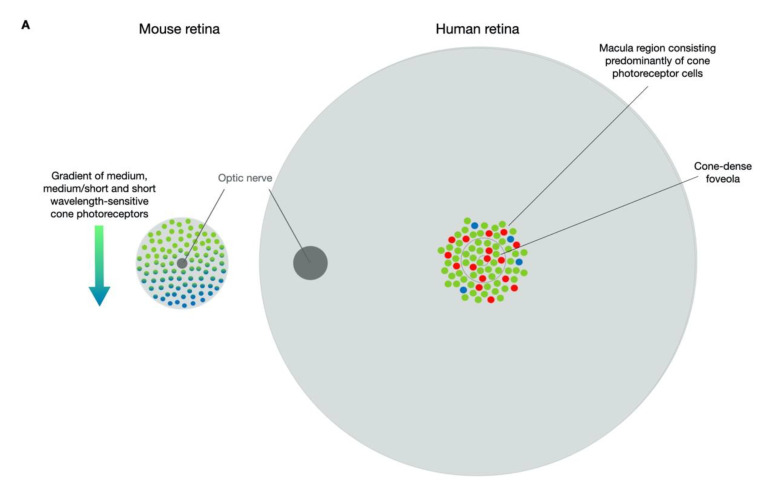

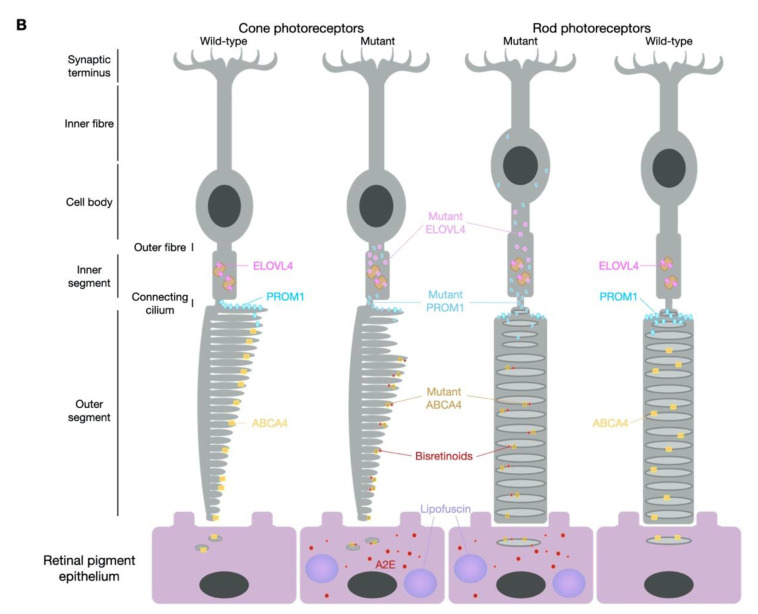
Comparisons of the human and mouse cone photoreceptor distribution and localisations of ABCA4, ELOVL4 and PROM1. (**A**) Rod photoreceptors comprise the majority of light-sensitive cells of the mouse retina with cone photoreceptors occurring in a decreasing gradient of medium-wavelength, medium/short-wavelength and short-wavelength sensitive varieties in a dorsal to ventral direction. By comparison, the human retina contains a macula region in which the foveola provides the site of maximum visual acuity and is densely packed with long- and medium-wavelength sensitive cone photoreceptors with short-wavelength sensitive cone photoreceptors occurring sporadically amongst the cones in the fovea centralis of the macula. Stargardt disease is characterised by a loss of cells in the cone-rich macula region. (**B**) In a wild-type state, ELOVL4 is located in the endoplasmic reticulum of photoreceptor cells, with mutant forms becoming mislocalised as well as losing function. PROM1 is typically located at the base of the outer segments where new discs are formed, and mutant PROM1 mislocalises within the photoreceptor cells and disc morphogenesis occurs. ABCA4 is present on the disc membranes and in mutant form causes dysregulation of the visual cycle, causing build-up of unwanted compounds in the disc membranes. When consumed by the RPE, these toxic compounds lead to lipofuscin accumulation and ultimately death of the RPE. ABCA4 = ATP-binding cassette transporter protein 4; ELOVL4 = elongation of very-long-chain fatty acids; PROM1 = prominin 1; RPE = retinal pigment epithelium.

The ABCA4 protein is a transport protein essential to the visual cycle and is located specifically in the outer segments of photoreceptor cells (Figure 1). It is responsible for moving retinoids, in particular *N*-retinylidene-phosphatidylethanolamine and phosphatidylethanolamine, via a flippase mechanism from the lumen to the cytoplasmic side of the disc membranes. This process removes retinoids from the photoreceptor discs and in so doing enables continuation of the visual cycle [5,6]. When this process is disrupted due to non-functional ABCA4, there is a build-up of retinoids in the disc membrane that leads to the formation of bis-retinoid fusion products, such as *N*-retinylidene-*N*-retinlyethanolamine (A2E) [7]. Photoreceptor cells constantly produce new disc membranes and as each new disc is formed, the older ones become more distal. The outer segments of photoreceptor cells are in contact with the supporting retinal pigment epithelium (RPE), one role of which is to phagocytose the distal discs. In a patient with STGD1 disease, the RPE cells consume the retinoid derivatives and as A2E is insoluble, it persists in these cells, where it has a tendency to aggregate and form lipofuscin [8]. This ultimately leads to cell damage and degeneration of the RPE cells and when the RPE cells die, the photoreceptor cells they support begin to lose function and also degenerate. In earlier stages of disease, the accumulation of bisretinoids throughout the cone cytoplasm may explain the early occult macular dystrophy phenotype that precedes cone dystrophy, long before RPE changes [9]. This review will focus on current and prospective treatments for STGD1, with further details on the function of ABCA4 to be found elsewhere [10]. Although mutations in ABCA4 had been proposed as risk factors for age-related macular degeneration (AMD) a decade ago [11], more recent extensive phenotype–genotype studies have defined late onset Stargardt disease as a distinct entity that appears clinically similar to AMD [12]. Hence, therapeutic interventions for ABCA4-related disease are unlikely to benefit a patient cohort beyond those with STGD1.

### 1.2. STGD3—ELOVL4 (Allelic to the Former STGD2)

ELVOL4 is also a membrane protein but is located in the endoplasmic reticulum and is involved in the biosynthesis of fatty acids [13]; in the retina expression appears to be limited to the photoreceptor cells [14]. Mutations in ELOVL4 not only lead to mislocalisation of ELOVL4, which has a negative impact on photoreceptor function [13], but also absence of enzymatic activity [15]. Photoreceptor outer segments containing mutant ELOVL4 protein also lead to phagolysosomal defects in the RPE, which further adds to the mechanism of disease [16]. Whilst autosomal dominant mutations in ELOVL4 cause STGD3, autosomal recessive mutations result in other conditions, including neuro-ichthyotic disease [17].

### 1.3. STGD4—PROM1

The *PROM1* gene, also known as CD133, has at least five alternative promoters that provide tissue-dependent expression [18] and is a membrane glycoprotein most commonly known for being a stem cell marker [19]. In the retina, the prominin 1 protein is typically found at the base of the outer segments, where it makes important associations with other components critical to disc morphogenesis [20]. It has also been shown that PROM1 regulates autophagy in the RPE, a process that protects the RPE from lipofuscin accumulation [21]. Autosomal recessive mutations cause retinitis pigmentosa [22,23], which is characterised by peripheral loss of vision, whereas autosomal dominant mutations cause STGD4 and other forms of macular dystrophy [23,24].

## 2. Models of Stargardt Disease

### 2.1. Mouse Models

A general issue when attempting to study diseases of the macula and assess the safety and efficacy of treatments, is that the favoured in vivo model, mice, have no macula. The murine retina is composed of ~95% rod photoreceptors with a decreasing cone photoreceptor gradient occurring from the superior to inferior retina (Figure 1). By contrast, the macula region in humans is cone-dense with the retina outside of this region composed predominantly of rod photoreceptors. Whilst many naturally occurring and transgenic mouse models reliably mimic human inherited retinal diseases, mutations in genes that cause macular degenerations regularly fail to generate desired phenotypes.

For STGD1, the *Abca4* knockout (KO) model has a complete absence of Abca4 protein. On an albino background carrying the homozygous Rpe65 Leu450Met mutation, the *Abca4* KO presents loss of photoreceptor cells with increasing age (by 11 months) [25]. However, on a pigmented background this does not occur [26] yet both *Abca4* KO models suffer from lipofuscin accumulation over time, which can be detected by the non-invasive technique of scanning laser ophthalmoscopy (SLO) and by direct quantification from post-mortem tissue by high-performance liquid chromatography (HPLC) analysis [27,28]. For SLO measurements, the 488 nm wavelength is considered to correlate to levels of A2E and the 790 nm wavelength to lipofuscin [28]. To better reflect a human genotype, a STGD1 mouse model was created carrying Abca4 Leu541Pro and Ala1038Val (PV) mutations [29]. The expressed double-mutant was shown to generate Abca4 protein at reduced levels compared to wild-type mice. As with the *Abca4* KO model, only age-related changes in structure were observed and there was comparable accumulation of A2E and lipofuscin over time. Similarly, a homozygous Abca4 Asn965Ser model also presented with reduced levels of Abca4 protein in the photoreceptor cells with mislocalisation evident in the inner segment structures [30]. Consistent with other STGD1 mouse models, A2E and lipofuscin accumulation occurred with age, evident by SLO fluorescence assessments and HPLC quantification. Whilst there tends to be a general absence of gross structural and functional changes in mouse models of STGD1, the accumulation of A2E and lipofuscin do enable measurements of treatment efficacy by the reduction in these compounds post-treatment.

Due to the roles of *Elovl4* in cell types other than the retina, homozygous *Elovl4* KO mice do not survive beyond birth [31]. Heterozygous *Elovl4* KOs develop normally with minimal structural or functional changes to the retina. The authors suggested that absence of the degenerative phenotype indicated that haploinsufficiency was not the mechanism for STGD3 disease. However, as previously mentioned, given the consistent problem in mimicking a macular disease in a mouse model, this was not fully supported until investigations into the retinal phenotype in the homozygous *Elovl4* KOs determined normal retinal development prior to death [32]. Further evidence ruling out *Elovl4* haploinsufficiency as the cause of STGD3 retinal degeneration came when transgenic and knock-in models were generated expressing disease-associated mutations [33,34,35]. Compared to previous models, these variants recapitulated human disease surprisingly well and exhibited loss of photoreceptor cells, abnormal function when measured by electroretinogram (ERG) and lipofuscin deposits in the RPE [36].

For investigations into disease pathology and treatment options for STGD4, there is a naturally occurring rd19 mouse model (http://www.informatics.jax.org/allele/MGI:5605699, accessed on 30 June 2021) in addition to transgenic [20] and *Prom1* KO [37] models. The naturally occurring rd19 model carries a premature stop codon (Lys269X) and exhibits photoreceptor loss by 2 months of age with associated abnormal rod ERG responses. Similarly, the homozygous *Prom1* null mouse also showed photoreceptor degeneration and is highly sensitive to light with rearing of the mice under dark conditions able to significantly preserve retinal structure and function [37]. For investigations of human relevant mutations, human *PROM1* knock-in mice were generated carrying the common Arg373Cys mutation, expressed specifically in rod photoreceptor cells [20]. In comparison to equivalent knock-in mice carrying the wild-type human sequence, mutant PROM1 expression caused mislocalisation of the protein and abnormal outer segment morphologies prior to degeneration.

### 2.2. In Vitro Models

Mouse models of disease provide an opportunity to investigate disease pathogenicity and treatment intervention, but the differences in phenotype can limit the usefulness of such models and there is therefore scope to expand pre-clinical testing to other models. Immortalised cell lines are commonly used to overexpress mutant protein forms and explore function. It is important to understand the expression profile, cellular localisation and function of a given mutant form in comparison to the wild-type variant in order to accurately evaluate the benefits of a subsequent treatment. Retinal genes are not often expressed natively in standard cell lines; therefore, exogenous DNA delivery is required. In addition to understanding the expression and localisation of mutant protein forms, information on the influence of a given mutation on function can also be achieved. In the case of ABCA4, this can be by way of ATPase activity [38] or for ELOVL4, the biosynthesis of very-long-chain fatty acids [15]. However, such in vitro experiments have their limitations. For example, assessing the impact of splice site mutations is challenging when using exogenous DNA delivery and can rely on midigenes (bacterially encoded splice intervals) that do not fully represent the final expression product [39]. If an in vitro system cannot reliably produce the mutated version of a protein for assessment of localisation, structure and function, evaluating subsequent therapy of the mutant to redeem the wild-type features could be limited. This has led to investigators exploring alternative models for in vitro assessments.

The generation of induced pluripotent stem cell (iPSC)-derived photoreceptor cells would be of great use to STGD1, STGD3 and STGD4 studies but this can be relatively technical [40]. Generating iPSCs can take 20–30 days, with the subsequent differentiation into retinal photoreceptor-like cells requiring a further 10–14 days of specialised culture conditions. As an alternative to this, a recent study presented evidence of *ABCA4* expression in cells isolated from human skin cells and hair follicles [41]. Comparison of hair follicles from control samples to those from a STGD1 patient carrying the mutation c.5836+2T > G revealed significantly lower *ABCA4* expression levels in the patient hair follicles. If hair follicles can be reliably used for assessment of *ABCA4* expression, then such samples might prove particularly useful in future testing of CRISPR-based mutation correction therapies (discussed later).

A further investment that would prove beneficial to the study of disease and treatment options for STGD1, STGD3 and STGD4 is the generation of retinal organoids from patient samples. After first generating iPSCs, differentiation into retinal organoids (an additional timeframe of 120–280 days depending on the degree of maturation desired) can provide evidence of disease phenotype, as has been seen with other such models for inherited retinal disease [42,43,44,45,46,47,48,49], and then provide an opportunity for correction of phenotype following treatment [42,45,48].

Whilst there is no single ideal model for assessing the treatment potential of therapeutic interventions for Stargardt disease, a combination of testing in the models discussed (Table 1) would prove highly informative. Model selection will depend both on the genetic origin and the treatment strategy being applied and whilst there is currently no approved treatment for Stargardt disease, there are numerous candidates showing potential. Given the relative prevalence of STGD1, the majority of treatment options discussed in this review will relate to ABCA4-related strategies, but STGD3 and STGD4 options will be considered where appropriate.

## 3. Gene Therapy for Stargardt Disease

With dozens of clinical trials ongoing and an approved gene therapy treatment for inherited retinal disease, the evidence of safety and efficacy of such an approach is building year on year. The focus to date has been on gene supplementation strategies for genes that cause autosomal recessive disease (for example, *RPE65* [50]) and X-linked disorders (for example, *CHM* [51] and *RPGR* [52]), which by their nature are amenable to such an approach. STGD1 would be considered a strong candidate for a gene supplementation strategy, but the large 6.8 kb coding sequence of *ABCA4* leads to difficulties in gene delivery, as the favoured vector is adeno-associated virus (AAV), which has an optimal packaging capacity of ~4.7 kb [53]. This has led to various strategies being developed to enable large gene delivery. By comparison, both *ELOVL4* and *PROM1* coding sequences are of a size that would package well into AAV, but as they are mainly autosomal dominant diseases with associated dominant negative mechanisms [23], gene supplementation would not likely resolve the disease condition. In the absence of generic pharmaceutical intervention, treatment options for STGD3 and STGD4 are therefore likely going to rely on future gene-editing technology (discussed later).

### 3.1. Lentiviral Vectors

Lentiviral vectors have been of interest for gene therapy due to their packaging capacity of ~8 kb, enabling them to carry even the largest coding sequences, including *ABCA4*. Equine infectious anaemia virus (EIAV) lentiviral vectors were shown to successfully transduce mouse [54] and macaque retina [55]. A full-length *ABCA4* coding sequence was packaged into EIAV-derived lentiviral vectors and delivered by subretinal injection into post-natal day 5 (P5) *Abca4* KO mice [56]. The transgene included a LacZ reporter and a correlation between ABCA4 protein expression and LacZ was achieved in treated eyes. Expression was sustained over 12 months and indications of a reduction in A2E accumulation were also achieved. It should, however, be noted that at P5, photoreceptors are still mitotic in the mouse retina and do not yet elaborate outer segment discs. These key anatomical features are likely to make it much easier for the cell-membrane enveloped lentiviral vectors to transduce immature photoreceptor cells compared with the adult, because the cell bodies of the latter are separated from the subretinal space by the dense lipid bilayers of up to 1000 compacted outer segment discs [57]. In a follow-up study, adult macaque and rabbit eyes were injected with GFP-EAIV or ABCA4-EAIV, respectively, without the LacZ reporter [55]. The EAIV lentiviral vectors were well tolerated in both species with only slight signs of inflammation in injected eyes. This became the StarGen vector (Sanofi, Oxford Biomedica, Oxford, UK), and in 2011 a Phase I/II clinical trial was initiated (NCT01367444). The trial was terminated in 2019 with study data on 27 patients reported online in June 2020 (https://clinicaltrials.gov/ct2/show/record/NCT01367444?cond=Stargardt+Disease&draw=2&rank=7, accessed on 30 June 2021). Serious adverse events were listed in two individuals, these being increased intraocular pressure in one and uveitis in another. All patients suffered one or more (not serious) adverse events but until the study is peer-reviewed, no conclusions can be drawn. With early termination of this study due to withdrawal of the sponsor Sanofi, all patients have been transferred to a 15-year follow-up (NCT01736592).

Despite their useful packaging capacity, the size and viral structure appear to limit the transduction capacity of lentiviral vectors in the retina [58]. With an absence of efficacy data from the clinical trial there is currently little encouragement for continued use of these vectors for Stargardt disease gene therapy. Whilst the interest in these vectors has waned in the last decade, the use of AAV vectors has accelerated.

### 3.2. Adeno-Associated Viral Vectors

Within the field of gene therapy, AAV vectors have been used extensively for therapeutic transgene delivery and in clinical trials for retinal disease they have shown good safety with minimal adverse events [59]. In the case of STGD1 treatments, early attempts involved creation of a single “oversized” transgene containing the complete 6.8 kb *ABCA4* coding sequence. When combined with other required transgene elements, this generated a transgene of over 10 kb. Attempts were made to package such oversized transgenes into AAV vectors and transduction of these preparations did enable production of the full-length protein both in vitro and in vivo [60]. However, the unregulated nature of the AAV packaging process meant that a heterogenous population of truncated transgenes were contained in the AAV capsids, leading to it being referred to as a fragmented dual-vector approach [61,62,63]. It was determined that the success of the approach came from AAV capsids containing transgenes that by chance happened to contain overlapping regions of sequence [64]. Assessments of a fragmented AAV–ABCA4 preparation revealed that in addition to forming the desired full length *ABCA4* transcript following recombination of overlapping truncated transgenes, hybrid transcripts of truncated fused *ABCA4* sequence were also present, of which some contained inserts of the AAV genome [65]. Despite the initial signs of success in attempting to package an oversized AAV transgene containing the complete *ABCA4* coding sequence, it became apparent that this approach was not going to be feasible due to the inability to reliably prepare homogenous AAV preparations containing defined transgenes. Given the anticipated treatment benefits of providing functional ABCA4, a shift was therefore made to dual AAV vector strategies.

Dual vector AAV strategies can take various forms, employing different mechanisms for formation of a single complete therapeutic element from two vectors [66]. One method uses the principle of overlapping regions that provided the success of the above fragmented strategy but with optimised and defined regions of coding sequence overlap [67,68] An alternative early approach was named *trans*-splicing and it provided no regions of overlap between the two transgenes but instead relied on the native tendency of transgenes to concatamerise via the inverted tandem repeat (ITR) elements that cap each end of a transgene [69,70]. Following joining of two transgenes via ITRs, splice donor and acceptor sites on either side of the fused ITR would enable removal of these extra sequences, leaving an intact full-length coding sequence. As with the overlapping dual vector approach, *trans*-splicing attempts to deliver full-length *ABCA4* showed early signs of success in mouse and pig models [67,71]. However, it is not possible to control concatamerisation; therefore, correct and incorrect orientations of concatamerised transgenes were achieved [72,73,74]. As a successful treatment would employ reliable mechanisms to enable success, the *trans*-splicing approach was combined with the overlapping principle to form a hybrid strategy [75]. This placed an overlapping sequence of homology between the splice sites on opposite transgenes, enabling reconstituted transgene formation by both the overlapping and *trans*-splicing mechanisms. Whereas the original overlapping strategy relied on using the coding sequence as overlap, hybrid dual vectors provided an opportunity to assess recombinogenic sequences of different origin [76,77].

All three dual vector strategies have successfully led to full-length ABCA4 expression in the *Abca4* KO mouse model and provided evidence of efficacy by way of reduced bisretinoid/A2E/lipofuscin accumulation [67,68,78]. One of the earlier studies initially suggested an overlapping strategy might not be viable for photoreceptor targeting [67]. Wild-type mice injected with AAV8 overlapping *ABCA4* vectors failed to produce full-length ABCA4 in eyes injected with 1 × 10^9^ genome copies per vector per eye when using either rhodopsin or rhodopsin kinase promoters (photoreceptor cell-specific promoters). However, 79% of injected eyes (11 of 14) did generate full-length ABCA4 when the ubiquitous CMV promoter was used. This led to a focus on *trans*-splicing and hybrid dual *ABCA4* vectors and it was found that when injected in albino *Abca4* KO mice and incorporating the rhodopsin promoter, 18% of eyes achieved full-length ABCA4 protein from the *trans-*splicing dual vector and 50% of eyes with the hybrid vector. Further to this, treated eyes showed a significant reduction in lipofuscin granules compared to untreated eyes. With the hybrid dual vector appearing to work best in this study, the researchers performed optimization in attempts to increase the efficacy [78]. Both aspects of hybrid dual vector recombination mechanisms were considered by comparisons of different ITR structures and the region of homology between transgenes. It was identified that homologous ITRs from the AAV2 genome were preferred for concatamerisation and that the F1 phage region of homology provided better recombination rates than an alkaline phosphatase sequence. This time, the rhodopsin kinase promoter was used with 2 × 10^9^ genome copies per vector per eye injected in wild-type mice. In this cohort, 50% of eyes generated full-length ABCA4 with variable expression levels evident between eyes. This was similar to the original findings and pigmented *Abca4* KO mice were subsequently injected with 1.8 × 10^9^ genome copies per vector per eye. At 3 months post injection the presence of lipofuscin in the RPE was measured using fluorescence intensity of eye tissue cryosections. The results suggest the RPE fluorescence intensity was significantly higher in untreated areas of retina than treated areas. These data were encouraging, but the fluorescence intensity measurement is difficult to standardise and since this study, more reliable methods of bisretinoid/A2E/lipofuscin accumulation have been employed, such as the previously mentioned SLO assessment of autofluorescence [28] and HPLC quantification of retinal tissue [27].

Despite the original lack of success with overlapping *ABCA4* dual vectors [67], it was considered that a viable strategy could still be obtained but that the success may lie in the region of homology between the two transgenes. A number of overlapping *ABCA4* coding sequence options were therefore compared, ranging from 1.17 to 0.09 kb in length with the optimal length identified to be 0.2 kb [68]. In addition to optimising the rate of recombination, truncated ABCA4 protein produced from the 3′ vector was successfully ablated by ensuring that the packaged coding sequence contained an out-of-frame “ATG” nucleotide sequence downstream of the 5′ITR prior to an in-frame “ATG” codon. The rhodopsin kinase promoter was used with transgenes packaged in the AAV8 Y733F serotype. Full-length ABCA4 was achieved in 90% of injected pigmented *Abca4* KO eyes when a high dose of 1 × 10^10^ genome copies per vector per eye was delivered. Protein lysate assessments indicated the total global levels of ABCA4 in injected eyes was 1–10% of that in wild-type eyes, yet in the region of injection, immunostaining suggested levels of ABCA4 in the photoreceptor outer segments were comparable to those of wild-type photoreceptors. Furthermore, in a blinded study, bisretinoid and A2E levels were compared by HPLC quantification of dual-vector-treated and paired sham-injected eyes 3 months post injection. Levels of bisretinoids/A2E were significantly reduced in the treated eyes, which aligned with the 790 nm (lipofuscin-related) SLO assessments that identified significantly less autofluorescence developed between 3 and 6 months post injection in dual-vector-treated eyes compared to paired sham-injected eyes.

A similar study used AAV8 Y733F to deliver a hybrid *ABCA4* dual vector system containing an alkaline phosphatase sequence for recombination [78]. Pigmented *Abca4* KO mice were injected with 3 × 10^9^ genome copies per vector per eye and at 4 weeks post injection full-length ABCA4 was detected at levels <20% of wild type. This study assessed relative A2E levels by 488 nm SLO measurements taken at 1, 2, 3, 4 and 5 months post injection. Levels of autofluorescence were significantly reduced in treated eyes compared to paired untreated eyes from 2 months post injection, which was maintained to 5 months post injection. At this latter time point, A2E levels were quantified by HPLC analysis and determined to be significantly reduced in the treated eyes.

A more recently described dual vector strategy involves intein-mediated reconstitution. Whereas overlapping dual vectors form the full-length *ABCA4* at the DNA phase and *trans*-splicing vectors at the RNA level, the intein strategy involves full-length ABCA4 reconstitution at the protein level [66]. Split N- and C-terminal intein polypeptides are added to the relevant extremities of each fragment of the ABCA4 protein. Upon interaction, the intein is removed by native cell processes and the two ABCA4 polypeptides combine to form the required therapeutic full-length protein. As with overlapping dual vectors, determining the optimal sequence and splitting point of the peptides will be crucial and likely gene/protein dependent [79]. One of the risks with this approach is that the tertiary structure and ultimate function of a given protein relies on post-translational modifications that may occur differently if two fragments are made separately and then combined. However, preliminary experiments have indicated highly encouraging results with full-length ABCA4 efficiently produced in retinal organoids and the treated eyes of mice and pigs [79]. Multiple splitting points for the *ABCA4* coding sequence were compared and the optimised *ABCA4-*intein dual vector system was injected into wild-type mice at 3.3 × 10^9^ genome copies per vector per eye. At 4–7 weeks post-injection, full-length ABCA4 protein (expression driven from the rhodopsin kinase promoter) was identified in 10 of 11 treated eyes, which also revealed high levels of truncated polypeptides from each single vector. An *Abca4* KO cohort injected with 4.3 × 10^9^ genome copies of the 5′ vector and 4.8 × 10^−9^ genome copies of the 3′ vector was assessed at 3 months post injection for lipofuscin accumulation. In this study, such measurements were made by lipofuscin granule counts by electron microscopy, which were significantly reduced in treated eyes compared to sham-injected eyes.

Collectively, these pre-clinical studies provide great encouragement for dual-vector strategies, although it should be noted that they vary in study design and post-treatment measurements in ways that make comparisons between efficacy data difficult to compare directly. For example, not all pre-clinical studies have compared treated eyes to sham-injected eyes. As a subretinal injection can cause a degree of change to the retina, it seems important to aim for a sham injection control. Additionally, measurements by SLO can be prone to bias, particularly in exposure levels between opposing eyes; therefore, conducting such assessments in a blinded manner should be strongly considered. An additional study feature could be to inject treatment and sham material into alternate contralateral eyes within a cohort to avoid further image bias.

Despite the variations in approach and specific methodologies used to measure post-treatment changes in bisretinoid/A2E/lipofuscin accumulation, the consistent success of the dual-vector strategies offer great hope for clinical trial initiation. All dual AAV vector systems carry risks of unwanted expression products, which have been evident in intein [79], overlapping, *trans*-splicing and hybrid *ABCA4* dual AAV vectors from both the 5′ elements [77,80] and the 3′ elements [68,77,81]. Prior to clinical trial, it needs to be shown that each component is safe as a single vector as well as when applied in combination, which so far has been presented for an overlapping *ABCA4* dual AAV system [82]. As described above, this dual-vector system was optimised to reduce/limit unwanted expression products [68], making each single-vector component inert until provided in combination. Such steps will be crucial for any dual AAV vector strategy before testing in humans.

A dual-vector system will inevitably be less efficient than a traditional single AAV strategy, achieving in the studies described above between 1 and 20% of wild-type levels of ABCA4 in treated eyes. However, whilst STGD1 onset occurs early in life, the rate of progression is typically slow. Understanding the biomolecular mechanisms of disease provides confidence that expressing any level of functional ABCA4 should be beneficial as it would reduce the rate of bisretinoid build-up that leads to retinal dysfunction and degeneration. The pre-clinical data now available provide encouraging signs that a dual-vector clinical trial for STGD1 could be worth pursuing.

### 3.3. Nanoparticles

Given the limitations of the viral vector delivery methods discussed, nanoparticles offer an alternative carrier for larger transgenes. Whereas viral vectors for inherited retinal disease may trigger an immune response [83,84], such responses would not be anticipated from artificial nanoparticles. Nanoparticles are cationic compounds that get wrapped in negatively charged DNA, typically in plasmid form, which provides a degree of protection from nuclease-mediated degradation whilst enabling passage through cell membranes by endocytosis or receptor-mediated uptake. The first nanoparticles used for ABCA4 delivery were polyethylene glycol-substituted polylysine (CK30PEG) with a packaging capacity range of 5–20 kb [85]. *Abca4* KO mice received subretinal injection of nanoparticles coated with plasmid carrying a complete *ABCA4* transgene containing either the interphotoreceptor-binding protein (IRBP) promoter or the mouse opsin promoter. Full-length ABCA4 protein expression was achieved up to 8 months post injection with peak expression observed at 2 months. Lipofuscin granules in the RPE were also reduced in treated *Abca4* KO eyes compared to untreated eyes. These data suggest that nanoparticles could deliver plasmid DNA to the photoreceptor cells of the retina and provide long-term expression of the desired therapeutic product. However, a further example of *ABCA4*-nanoparticle delivery to photoreceptor cells was not achieved until more recently [86]. A new preparation of pH-sensitive amino lipid nanoparticles was used of (1-aminoethyl)iminobis [N-(oleoylcysteinyl-1-amino-ethyl)propionamide) (ECO), which not only self-assemble with the DNA but also enable amphiphilic endosomal escape and reductive cytosolic release [87,88]. Pigmented *Abca4* KO mice were injected with ECO nanoparticles carrying compact plasmid DNA containing the bovine rhodopsin (RHO) promoter with the full-length *ABCA4* coding sequence. At 4 days post injection, levels of *ABCA4* mRNA were 500–2500 fold greater than untreated eyes but by 4.5 months post injections, *ABCA4* mRNA levels were 2–15 fold above background. No evidence of ABCA4 protein expression was provided but there was an indication of reduced A2E accumulation in ECO-RHO.ABCA4 treated eyes relative to untreated eyes 8 months post injection. No signs of toxicity were evident, but this was not specifically assessed in the study and needs further investigation. Given the encouraging recent attempts to enhance and optimise the formulation [89], these ECO nanoparticles seem a promising approach to pursue for supplementation of ABCA4.

### 3.4. Anti-Sense Oligonucleotides

Anti-sense oligonucleotides (AONs) are small, single-stranded fragments of artificial nucleotides that act as RNA modulators [90]. They bind to complementary nucleotides of either mRNA or pre-mRNA, enabling prevention of splicing at deep intronic premature splice sites or to enforce exon skipping to prevent toxic protein production. Their potential use for the treatment of Stargardt disease is endorsed by data from the ongoing clinical trial (NCT03140969). Patients with the deep intronic mutation c.2991 + 1655A > G in *CEP290*, which causes the inherited retinal dystrophy Leber congenital amaurosis, have so far received an intravitreal injection of sepofarsen (ProQR Therapeutics), a 17-mer 2′-O-methyl-modified phosphonothioate RNA AON, followed by three monthly top-up injections. Importantly, no serious adverse events have been reported with encouraging signs of improvements to visual acuity at 3 months post treatment [91]. More recently, it was revealed that one patient achieved a sustained response to just a single dose of sepofarsen lasting up to 15 months after treatment [92]. These clinical trial data suggest that such intervention in Stargardt patients could also be achievable. Prior to the sepofarsen clinical trial, there were some concerns about delivering AONs to the eye regarding potential inefficient uptake and degradation. However, pre-clinical assessments consistently showed the precursor to sepofarsen was taken up by cells and able to reduce levels of the mutant *CEP290* variant [93]. Structural modifications have since occurred to improve the efficacy, with the clinical trial data proving the safety and success of the strategy.

An AON therapeutic strategy is not likely to be relevant for STGD3 as *ELOVL4* mutations identified to date cause loss-of-function or dominant-negative changes to the protein [94]. However, STGD4 can arise due to splice site mutations and indeed a deep intronic *PROM1* mutation caused by pseudoexon activation has been described [95] and would be amenable to an AON strategy. However, it is STGD1 that will have the largest patient cohort for AON treatment and there have been multiple reports of deep intronic mutations that respond to AON therapy in pre-clinical testing. This has been achieved in photoreceptor precursor cells generated from patient skin biopsies [96,97,98] and with midigene assays in HEK293T cells [98,99].

Another use of AONs for gene therapy has emerged by way of endogenous adenosine deaminase acting on RNA (ADARs) [100]. These enable mutation-specific base editing to reverse guanosine > adenosine mutations, performing the adenosine > inosine transition with inosine then read as guanosine. There are two known endogenous ADARs: ADAR1 and ADAR2. ADAR1 has two isoforms and is ubiquitously expressed but is limited to editing non-coding and repetitive regions of sequence [101]. ADAR2 has one predominant isoform and is the variant harnessed for base-editing interventions as it targets coding regions [102]. It has been shown that ADAR2 RNA is expressed in the retina with immunohistochemical staining identifying ADAR2 in the retinal ganglion cells but as yet expression in other cell types has not been revealed [103]. Given that *ABCA4*, *ELOVL4* and *PROM1* are all expressed in the photoreceptor cells, recruitment of endogenous ADAR2 can only be feasible if it is expressed in these cells, whereas ADAR1 may be useful for targeting intronic variants. Different RNA structures for recruiting endogenous ADARs have been assessed, varying from long RNA (71–191 nucleotides) for the Leveraging Endogenous ADAR for Programmable Editing of RNA (LEAPER) system [104] to the shorter 20–40 chemically modified nucleotides used for the Recruiting Endogenous ADAR to Specific Transcripts for Oligonucleotide-mediated RNA Editing (RESTORE) system [100]. It is also possible to attract endogenous ADAR by incorporating recruitment domains [105]. Whilst there is potential in an RNA base-editing strategy for Stargardt disease [4,106], until more is known about the endogenous ADAR expression profile in the retina, it may be that co-delivery of base editors with guiding RNA sequences will be required (see later discussion).

## 4. Small Molecule Therapy for Stargardt Disease

### 4.1. Pharmaceutical Interventions

Small molecule therapies enable targeting of a particular step in the visual cycle or aspect of retinal function that is altered by Stargardt disease. Whilst these are not likely to be curative, they aim to reduce symptoms and inhibit progression of the disease. Given the complexity of the visual cycle and the differing roles of ABCA4, ELOVL4 and PROM1, finding suitable generic candidates has proven difficult. However, there are currently multiple clinical trials involving compound therapies that intervene in key pathological pathways that occur in Stargardt disease.

**Figure 2:**
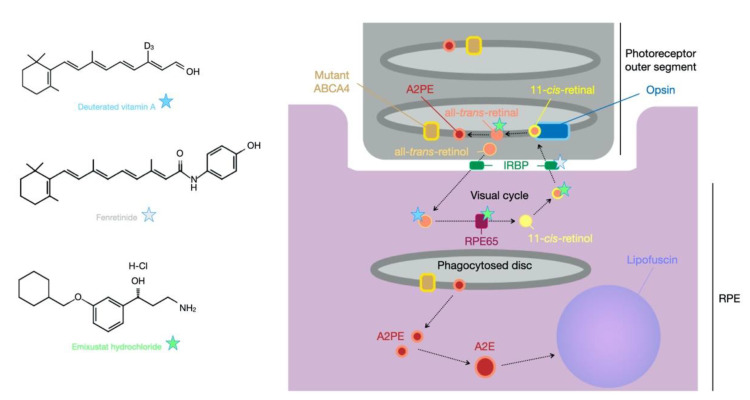
Example pharmaceutical interventions for Stargardt disease. Treatment strategies for STGD1 include pharmaceutical products that interfere with the biochemical pathways that lead to the disease state. IRBP = interphotoreceptor retinoid-binding protein; RPE = retinal pigment epithelium; RPE65 = retinoid isomerohydrolase.

As described earlier, in the case of STGD1, mutations in ABCA4 result in a non-functional protein, thereby limiting or abolishing the normal transport of *N*-retinylidene-phosphatidylethanolamine. This leads to build-up of all-*trans*-retinal in the intradiscal space in addition to reducing the clearance rate of *N*-retinylidene-phosphatidylethanolamine (NretPE), which causes the generation of A2PE in the disc membranes. The combination of these events leads to generation of *N*-retinylidene-N-retinylethanolamine (A2E), a major component of lipofuscin that accumulates in the cells of retinal pigment epithelium following disc phagocytosis (Figure 2) [107]. The majority of ongoing clinical trials aim to influence this process in various ways. For example, emuxistat hydrochloride is a visual cycle modulator that is a direct inhibitor of the visual cycle component retinol isomerase RPE65 [108] and in so doing slows the regeneration of 11-*cis*-retinal, reducing production of retinaldehyde, but it has also been shown to sequester the cytotoxic all-*trans*-retinal [109]. Patients with confirmed mutations in *ABCA4* were recruited for a Phase IIa trial in 2017 (NCT03033108) and received either a daily dose of 2.5, 5 or 10 mg oral emuxistat for one month. Delayed dark adaption was reported in 47% of participants, occurring most frequently in the 5 (67%) and 10 mg (57%) cohorts. Suppression of the rod photoreceptor b-wave was therefore dose dependent and confirmed the biological activity of emuxistat [110]. This delayed dark adaptation was a consequence of the drug’s mechanism of action and not a treatment effect, but with the absence of other adverse events a Phase III trial was initiated that will investigate the efficacy of the 10 mg dose in STGD1 patients (NCT03772665). It is worth noting that emuxistat trials have also occurred for patients with age-related macular degeneration, but data following a 24 month randomised clinical trial revealed emuxistat did not reduce the growth rate of geographic atrophy in such patients. However, given the complex, late-onset and multifactorial nature of the disease, this does not suggest that such trials in STGD1 patients will not be effective.

The build-up of A2E and other bisretinoids can lead to activation of the complement system in RPE cells [111] and complement inflammatory markers have been identified as elevated in the *Abca4* KO model [112]. Complement activation is highly involved in the aetiology of age-related macular degeneration [113], and given the shared features between age-related macular degeneration and STGD1, drugs designed for one may be relevant to the other. Avacincaptad pegol (Zimura) is an anti-C5 aptamer that aims to prevent or reduce the destructive effects of the activated complement cascade. Avacincaptad pegol has been provided as an intravitreal injection to Phase IIb clinical trial patients (NCT03364153); recruitment of 120 STGD1 patients began in 2018 with results yet to be reported. However, data from a Phase II/III trial in 286 patients with age-related macular degeneration determined the drug was well tolerated with evidence of efficacy achieved. Indeed, a significant reduction in the growth rate of geographic atrophy (by 27%) was observed in patients that received either 2 or 4 mg doses [114]. These encouraging data enable a degree of optimistic anticipation for the forthcoming results of the ongoing trial for STGD1. Another drug, eculzimab, has been explored for C5 inhibition but this is an IgG antibody, and though well tolerated in a Phase II clinical trial (NCT00935883), no signs of efficacy were achieved in patients with age-related macular degeneration [115] and there appear to be no indications of further trials in other cohorts.

A common target of the visual cycle is vitamin A (all-*trans*-retinol), which is a precursor to 11-*cis*-retinol. It is postulated that reducing levels of vitamin A could limit the production of all-*trans*-retinal/PE and subsequently A2E. Whilst supplements of standard vitamin A may be detrimental, as observed in the *Abca4* KO model [25], an alternative approach has been the provision of deuterated vitamin A (ALK-001). This drug has the C20 hydrogen atoms replaced with deuterium atoms, an isotope of hydrogen with a neutron in the nucleus, Figure 2. This impedes the dimerization of vitamin A and therefore reduces the opportunity for production of A2E [116]. Dietary provision of C20-deuterated vitamin A has been shown in *Abca4* KO mice to prevent the disease phenotype [117]. The tight dietary control required to achieve these results in mice may not be feasible in humans, but a Phase I clinical trial (NCT02230228) has at least shown oral ingestion of C20-deuterated vitamin A to be safe. A Phase II trial for STGD1 patients is ongoing (NCT02402660) with preliminary data presented at the 2019 ARVO Annual Meeting [118]. It was considered that 90% of dietary vitamin A intake had been replaced by deuterated vitamin A with no unexpected adverse events reported. No data relating to efficacy have been provided to date, but a new clinical trial has been initiated to extend the assessments of tolerability and efficacy of ALK-001 (NCT04239625). Another vitamin A-related treatment potential for STGD1 is fenretinide (Sirion Therapeutics), which is a synthetic derivative of vitamin A that binds retinol-binding protein and reduces the circulating levels of this protein, which in turn decreases levels of vitamin A and subsequently A2E in *Abca4* KO mice [119]. Whilst no trials have been initiated with STGD1 patients, a Phase II trial of 246 patients with age-related macular degeneration (NCT00429936) identified adverse events in 20% of the high-dose cohort (300 mg daily oral dose) for whom treatment was stopped. Serum levels of retinol-binding protein were reduced in a dose-dependent manner, but evidence of efficacy was not significant [120]. The drugs A1120 and STG-001 (Stargazer Pharmaceuticals) are also inhibitors of retinol-binding protein. In contrast to fenretinide, A1120 is a non-retinoid retinol-binding protein antagonist that reduced the accumulation of lipofuscin in the *Abca4* KO model with no significant impact on ERG [121]. It was shown not to act as a retinoic acid receptor alpha agonist, which may potentially improve its safety profile in comparison to fenretinide, but until clinical trials are attempted, this is difficult to infer. Minimal information is available on the drug STG-001 other than the knowledge that it is an inhibitor of retinol-binding protein and for which a safety trial in healthy individuals has been completed (ACTRN12619000816156) with no release of data. However, a subsequent trial for STGD1 patients was initiated in July 2020 with no findings yet presented (NCT04489511).

### 4.2. Dietary Supplementation

Other small-molecule clinical trials ongoing for Stargardt disease involve dietary supplements. Docosahexaenoic acid (DHA), eicosapentaenoic acid (EPA) and alpha-linolenic acid (ALA) are omega-3 fatty acids. DHA is the major very-long-chain polyunsaturated fatty acid of the retina and is a particularly critical component of photoreceptor cells [122]. Dietary DHA supplementation was investigated in STGD1 and STGD3 patients to determine whether macular function could be improved (NCT00060749). Trial data relating to the STGD1 patients recruited have been reported: patients received DHA supplementation (2000 mg/day) followed by 3 months of placebo, which was repeated to form a 12-month period with no improvement in macular function observed in the small cohort recruited (11 subjects) [123]. This STGD1 cohort was recruited on the hypothesis that dietary DHA supplementation may cause non-specific effects and would therefore be used as a control sample for the STGD3 cohort, data for which have yet to be revealed. Pre-clinical assessments suggested DHA supplementation had a positive effect on retinal function in heterozygous *ELOVL4* knock-in STGD3 mice [35] at 6–12 months of age and preservation of cone function was also achieved in 12–18-month-old wild-type mice [124]. The MADEOS trial (Macular Degeneration Omega-3 study, NCT03297515) is a further trial comparing dietary supplementation of omega-3 fatty acids against a placebo of sunflower oil and will include patients with age-related macular degeneration and Stargardt disease.

A final dietary supplementation used in clinical trials in attempt to counteract oxidative stress that can induce the accumulation of lipofuscin, is saffron (NCT01278277). A total of 31 patients with *ABCA4* mutations were randomly assigned to two groups and consumed either 20 mg oral saffron daily for 6 months followed by a switch to the placebo for 6 months (14 patients) or vice versa (17 patients). Macular cone-mediated responses were assessed with no significant findings achieved after 6 months [125]. However, it may be that assessments over a longer period of study would achieve more indications of treatment effect and measurements of autofluorescence were not acquired, which would be a further outcome measure worth pursuing.

## 5. Cell Replacement Therapy for Stargardt Disease

With atrophy of RPE and photoreceptor cells causing loss of visual acuity in patients in later stages of disease, the treatment options considered up to this point would only be beneficial for preventing further sight loss. In order to regain lost vision, transplantation of new cells would be required. Pre-clinical studies providing human embryonic stem cell (hESC)-derived RPE cells into the subretinal space of mice have provided encouraging proof-of-principle data. The hESC-RPE cells were transplanted into the Royal College of Surgeons rat model, which suffers vision deterioration over time due to a mutation in *Mertk*, a c-mer proto-oncogene kinase receptor specific to RPE cells [126]. Non-functional Mertk causes RPE dysfunction by preventing phagocytosis of shed photoreceptor outer segment discs, which leads to death of the photoreceptor cells. In the RCS rats, optometer responses were used as measurements of visual acuity and animals that received 50–100,000 cells performed significantly better than untreated and sham-injected animals [127]. The hESC-RPE cells were also injected into an STGD3 mouse model with a marginal significant improvement in optomoter responses observed in cell-treated eyes compared to sham-injected and untreated controls at 5 weeks post surgery. These data led to Phase I/II clinical trials (NCT01625559 and NCT01345006) in which late-stage patients with Stargardt’s disease received hESC-RPE transplantation. Importantly, no serious adverse events relating to the transplanted cells were reported and best corrected visual acuity was increased in transplanted eyes compared to non-transplanted control at 12 months after surgery, although the sample size was too small to determine significance [128]. There have been several similar trials, including NCT01469832, in which 12 STGD1 patients received escalating doses of hESC-RPE cells. Whilst no detrimental effects were identified, improvements in retinal function and visual acuity were not apparent [129]. Follow-up trials to these are ongoing (NCT02445612 and NCT02941991) and will be of particular interest to confirm the safety of such intervention.

A recent report presented data from 17 Stargardt disease patients that received autologous (patient-derived) bone-marrow-derived stem cells (BMSC) in both eyes (NCT01920867) [130]. Whilst the study was open to STGD1, STGD3 and STGD4 patients, the genetic mutation in the patients reported was not specified. The study remains highly controversial because participants had to pay a significant sum to be in the trial, which is against the internationally agreed research ethics guidance defined by the World Medical Association (WMA) in the Declaration of Helsinki. Furthermore, by recruiting only patients who believe the treatment will benefit them and without having any sham-injected controls, the study will be hugely biased towards a placebo effect. This is not to say BMSC transplantation might not be effective, but the study design and recruitment process of a trial should meet international standards.

A key consideration with RPE transplantation for Stargardt disease is that it does not directly approach the cause of the disease, and therefore may not enable long-term effects. Providing RPE cells back into a retina that has lost these cells and the photoreceptor cells in the same area may not be the most therapeutic transplantation option given that in patients with Stargardt disease, the problem arises in the photoreceptor cells. Provision of new iPSC-derived RPE cells may only be a short-term solution, particularly as these cells will be prone to A2E and lipofuscin accumulation, as were the original RPE cells [131]. The clinical trial data for the studies presented here are highly important for determination of the safety of such procedures and may lead the way for other cell transplantation interventions. For example, ReNeuron are undertaking a Phase I/IIa trial in patients with retinitis pigmentosa who will receive a subretinal transplant of human retinal progenitor cells (NCT02464436). If the safety of such transplantation is shown then it may be that future trials could involve patients with Stargardt disease. It is also possible that in the near future, gene-editing correction of patient iPSCs will be performed with subsequent differentiation into retinal progenitor cells that can be transplanted back into the patient [132].

## 6. Future Therapy Prospects—CRISPR

**Figure 3:**
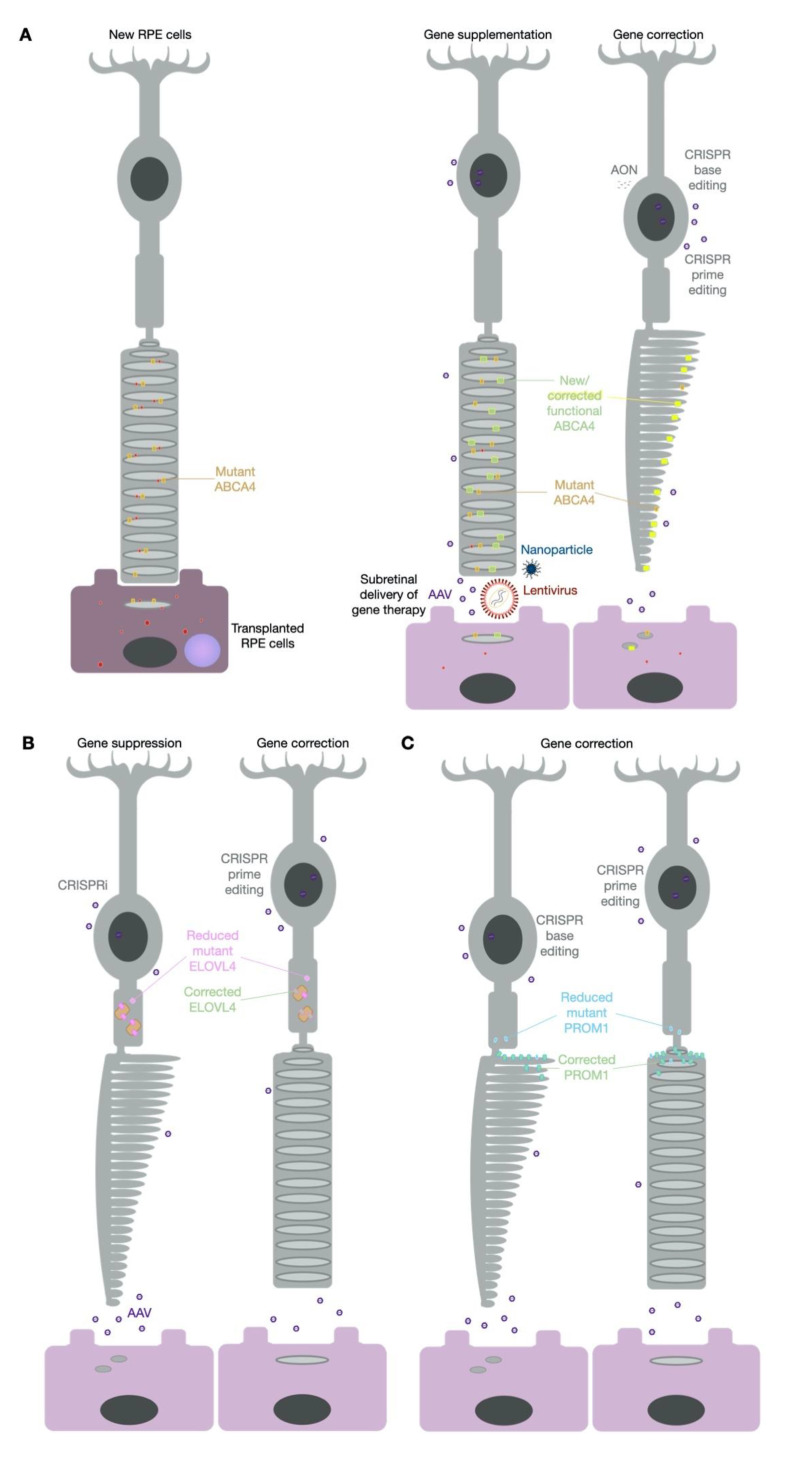
Comparisons of therapy strategies for Stargardt disease. Transplantation of RPE cells has been performed in STGD1 patients with gene supplementation and CRISPR-based genome-editing vectors as future treatment options (**A**). Therapies for STGD3 (**B**) and STGD4 (**C**) will likely focus on CRISPR-based approaches.

It is apparent that researchers have taken multiple approaches to the problem of vision loss caused by Stargardt disease (Table 2). Many of the treatment options considered show great potential, yet it may be that the most effective therapy is yet to be developed (Figure 3). Much has been written recently about the potential of clustered regularly interspaced short palindrome repeat (CRISPR)-based molecular tools and, in particular, their application for the treatment of inherited retinal disease [133,134,135]. Given the numerous reviews on this topic it is not necessary to discuss the mechanistic details here, but suffice to say the discovery of CRISPR has expanded treatment potential for currently untreatable diseases. With ever increasing discoveries of new Cas proteins from various bacterial origins and the development of fusion proteins to expand the molecular functions of these proteins, a new era of gene therapy has begun. In the case of Stargardt disease, a large proportion of mutations in *ABCA4, ELOVL4* and *PROM1* could be targeted with one or more of the currently described CRISPR-based approaches: genome editing, epigenetic repression, base editing or prime editing (Table 3).

The original form of CRISPR came by way of active Cas9, which targets DNA and leads to random insertion or deletion (indel) creation that can be used for gene silencing [136]. Alternatively, inactive Cas9 can achieve epigenetic repression known as CRISPR interference (CRISPRi) without needing to edit the DNA sequence [134]. Indel creation is currently being utilised in the EDIT-101 clinical trial to prevent cryptic exon and premature truncation of CEP290 in Leber congenital amaurosis patients carrying the mutation c.2991 + 1655A > G [137]. The outcomes of this first-in-human use of genome editing by CRISPR-Cas9 for an inherited retinal disease will be of great interest in determining the safety of this new form of gene therapy. Due to the nature of the mutation within an intron, direct gene disruption to remove the premature splice junction appears to be an effective strategy for this type of mutation [138]. As gene disruption by indel formation is an uncontrolled event in terms of the edits that arise, such an approach for targeting an exonic mutation would be restricted to an autosomal dominant disorder for which one wild-type copy of a gene would be sufficient to maintain the non-disease state. This would also be relevant for CRISPRi silencing of a mutant allele and *ELOVL4* may be an appropriate candidate for such a strategy. As considered earlier in this review, haploinsufficiency is not the underlying mechanism of STGD3, which suggests that silencing production of the mutant form of *ELOVL4* may offer therapeutic potential.

STGD4 is an autosomal dominant disorder that predominantly arises due to the mutation c.1117C > T (p.Arg373Cys) in *PROM1*, although two other missense mutations have also been identified [139,140]. The most common mutation of c.1117C > T would confer a G > A change on the complementary strand. This creates an opportunity for a DNA adenine base editor (ABE) to target the complementary strand and perform an A > G transition, thus correcting the “T” back to a “C” on the reading strand. Cas9–ABE fusion proteins are currently showing potential at such targeted editing and indeed the *PROM1* c.1117C > T site occurs within a good editing window for targeting by Cas9 proteins from *Streptococcus pyogenes* (SpCas9 [141]), *Staphylococcus auricularis* (SauriCas9 [142]) and a modified variant from *Staphylococcus aureus* (KKH-SaCas9 [143]). This makes the predominant STGD4 variant potentially treatable by CRISPR base editing. The current limitations of such constructs include their size, as the Cas9 element fused to the base editor make packaging the therapeutic construct into a single AAV vector difficult. However, lentiviral [144] and dual vector solutions are being used successfully [145,146,147] for delivery of CRISPR constructs as well as nanoparticles [148]; therefore, the gene therapy delivery systems discussed earlier in this review are also relevant to CRISPR therapeutics.

Base editing is also a viable strategy for STGD1. Patients can carry more than one *ABCA4* mutation, but as an autosomal recessive condition, the correction of one allele should be enough to provide a therapeutic benefit [149]. However, if two mutations are on the same allele, effective treatment will require the targeting of both. Furthermore, there are hundreds of pathogenic mutations confirmed in *ABCA4* (Table 3) and only a subset will be amenable to CRISPR base editing due to the requirement for a protospacer adjacent motif (PAM) sequence upstream of the mutation. In addition to the A > G correction by adenine base editors fused to Cas9, there are also base editors that enable C > T transitions [150]. This enables not only correction of T > C mutations but also A > G mutations that can be corrected by targeting the complementary strand. Of all the current pathogenic *ABCA4* mutations, 63% are considered to be editable transition variants and indeed correction of the five most common variants would be beneficial for 21% of patients [4]. Despite the encouraging findings of other STGD1 treatment options being explored, CRISPR base editing appears to be a viable avenue to pursue.

For G > A mutations, a further option is to attempt site-directed RNA base editing with adenosine deaminase acting on RNA (ADAR) enzymes fused with an RNA-targeting Cas protein such as Cas13 [106]. Whilst limited to corrections of G > A mutations, the benefit of RNA editing is that it does not directly alter the native DNA and that any edits are transient for the lifetime of the mRNA molecule. In this respect, RNA editing may offer a safer option for correction of G > A mutants. In addition to this, there is no PAM site requirement for Cas13; therefore, in theory, any “A” can be converted to a “G” by this approach. Both RNA and DNA base-editing systems currently suffer from bystander activity, in which nearby “A” can be converted to “G”, potentially altering the coding sequence and producing a new mutant variant in the attempt to correct one. However, the field is advancing rapidly and new refined options are developing at a quick rate [151], making base editing a likely therapeutic option for Stargardt disease in the near future.

Prime editing is a further CRISPR-based approach worth considering that targets DNA and consists of a Cas9 fused to an engineered reverse transcriptase [152]. In combination with a specially designed prime-editing guide RNA, this enables it to remove mutations and surrounding nucleotides and replace them with corrected sequence. Based on this mode of action, it is not limited to single base transitions but can also resolve transversions, insertions and deletions. With its many complementary components, the on-target editing rates currently appear low compared to other CRISPR approaches, but off-target and bystander editing rates appear reduced and the PAM requirement less stringent [152,153]. This is a new and emerging CRISPR tool for gene therapy with few reports published so far, but given its potential in correcting the vast majority of mutations that cause Stargardt disease, including the most common STGD3 5 bp deletion in *ELOVL4* [14], it is an incredibly exciting option for the future.

## 7. Concluding Remarks

Despite the large cohort of Stargardt disease patients, in particular those with STGD1, development of an effective treatment has lagged behind other rarer recessive inherited diseases. As discussed in this review, researchers and clinicians have approached the problem from multiple directions with many therapeutic strategies showing promise. Indeed, some of the options that have shown pre-clinical success have yet to be applied in humans. For all the encouraging therapeutics tested in trials to date, it may be that the most effective treatment form is yet to come by way of CRISPR-based strategies. Regardless of the approach employed, it is clear that with the efforts made so far, there is hope an effective treatment for Stargardt disease will appear in the coming years.

## Figures and Tables

**Table 1:** Summary of experimental models relevant for pre-clinical studies of Stargardt disease therapies. A2E = *N*-retinylidene-*N-*retinylethanolamine; ABCA4 = ATP-binding cassette transporter protein 4; ELOVL4 = elongation of very-long-chain fatty acids 4; ERG = electroretinogram; KO = knockout; ONL = outer nuclear layer; PROM1 = prominin 1; Rd19 = retinal degeneration model 19; RPE = retinal pigment epithelium.

Model Type	Details	Structural Features	Functional Features	Strengths/Limitations
Mouse				
STGD1	*Abca4* KO [25,26]	Absence of Abca4 expression. On an albino background, loss of outer nuclear layer (ONL) structure at 11 months. Pigmented mice show no loss in structure. Lipofuscin granule accumulation in the RPE.	*Abca4* KO models exhibit increased autofluorescence compared to age-matched wild-type mice that correlates to accumulation of bisretinoids/A2E/lipofuscin.	Easy detection of ABCA4 protein following gene supplementation. Assessment of pharmaceutical, dietary and gene therapy efficacy achievable by reduction in autofluorescence and associated build-up of bisretinoids/A2E/lipofuscin. However, the KO genotype and absence of Abca4 does not reflect typical human disease.
STGD1	Leu451Pro and Ala1038Val (PV/PV) [29] Asn965Ser [30]	Reduced expression of Abca4 with mislocalisation within the photoreceptor cells.	Models exhibit increased autofluorescence compared to age-matched wild-type mice that correlates to accumulation of bisretinoids/A2E/lipofuscin.	Efficacy evident in these models would be more relevant to human disease and achieved by rescue of bisretinoid/A2E/lipofuscin build-up and the associated autofluorescence phenotype.
STGD3	*Elovl4* KO [31,32]	Normal retinal structure.	Normal retinal function.	The KO is of limited value as it can only be reared as a heterozygous model and offers no clear features of retinal disease.
STGD3	*ELOVL4* 5-bp deletion knock-in [33,34,35,36]	Accumulation of ELOVL4 at 4 months with progressive loss of ONL and, in particular, cones at 6–18 months.	Abnormal ERG and accumulation of lipofuscin.	Rescue of retinal structure and function. Transgenic models are more representative of human disease both in genotype and phenotype.
STGD4	*Rd19*	Progressive loss of ONL beginning at 2 months of age.	Normal cone ERG but abnormal rod a-wave responses.	This naturally occurring model has yet to be used in pre-clinical studies.
STGD4	*Prom1* KO [37]	Extensive loss of ONL beginning at 2 weeks of age.	Abnormal ERG.	Loss of retinal structure and function begins early; therefore, treatment intervention may not be provided in time to observe efficacy. Rearing in the dark could be applied to slow the rate of degeneration.
STGD4	*PROM1* Arg373Cys knock-in [20]	Mislocalisation of PROM1with abnormal outer segment morphology and degeneration.	Abnormal rod and cone ERG by 3 months of age.	The knock-in better reflects the human state and offers an opportunity to assess treatment efficacy through correction of structural and functional changes.
In Vitro				
Immortalised cell lines	Wild-type	Lack of native retinal gene expression and absence of specialised retinal structures.	Enables expression and localisation assessments plus downstream isolation and functional assays.	Exogenous delivery of retinal genes of interest is required but basic assessments of vectors and downstream functional assays are achievable.
Induced pluripotent stem cells (iPSCs)	Patient-specific genotype	Cells can be differentiated to better reflect photoreceptor cell morphology and gene expression profiles.	Functional outputs could be achieved by expression profile analysis and downstream protein isolation and functional assays.	These will be particularly useful for future gene-editing techniques in assessing mutation-specific therapies. Editing efficiencies and protein outputs could be compared to cells from control donors.
Fibroblasts	Patient-specific genotype	Some retinal gene expression may be evident, as for *ABCA4* [41].	Functional outputs could be achieved by expression profile analysis and downstream protein isolation and functional assays.	The use of these will likely be supplementary to preliminary pre-clinical assessments of new therapies as expression of retinal genes will be limited. However, being patient-derived, they will have the added benefit of being useful for gene-editing strategies.
Hair follicles	Patient-specific genotype	Some retinal gene expression may be evident, as for *ABCA4* [41].	As for fibroblast samples, functional outputs could be achieved by expression profile analysis and downstream protein isolation and functional assays.	As for fibroblast samples, the use of these will likely be supplementary to other preliminary pre-clinical assessments but being patient-derived they will have the added benefit of being useful for gene-editing therapies.
Retinal organoids	Patient-specific genotype	Structural differences may be evident and include protein mislocalisation [42,43,44,45,46,47,48,49].	As for other patient-derived samples, functional outputs could be achieved by expression profile analysis and downstream protein isolation and functional assays.	Changes in expression profiles and protein localisation plus cell morphology could be assessed following treatment application. Retinal organoid will provide an ideal model for mutation-specific treatments.

**Table 2:** Summary of therapeutic approaches for Stargardt disease and related clinical trials. AAV = adeno-associated virus; AMD = age-related macular degeneration; AON = anti-sense oligonucleotide; BMSC = bone-marrow-derived stem cells; CRISPR = clustered regularly interspaced short palindromic repeats; DHA = docosahexaenoic acids; ESC = embryonic stem cells; N/A = not applicable; RPE = retinal pigment epithelium.

Strategy	Therapeutic	Trial	Phase	Data
Gene-based				
Gene supplementation	Lentivirus	NCT01367444	Terminated in 2019 due to sponsor issues, not for reasons of safety.	Yet to be peer-reviewed.
		NCT01736592	Follow-up of patients involved in the above trial.	
Gene supplementation	Dual AAV	N/A	Trials have yet to be initiated.	
Gene modulation	AON	N/A	N/A	
Gene editing	CRISPR	N/A	N/A	
Pharmaceutical				
Visual cycle modulator	Emixustat hydrochloride	NCT03033108	Phase I/IIa (completed 2021)	Delayed dark adaptation at 5 and 10 mg doses confirmed biological activity of the drug [110].
		NCT03772665	Phase III (initiated 2018)	Data not yet reported.
Deuterated vitamin A	ALK-001	NCT02230228	Phase I (completed 2015)	Therapeutic was well tolerated.
		NCT02402660	Phase II (initiated 2015)	Tolerability data were reported at ARVO 2019 [118].
		NCT04239625	Phase II (initiated 2020)	This is an extension of the above study of tolerability, safety and efficacy.
Inhibitors of retinol-binding protein	Fenretinide	No STGD trials	N/A	Adverse events in 20% of AMD patients at the high dose with no signs of efficacy.
	A1120	None reported	N/A	
	STG-001	NCT04489511	Phase IIa (completed 2021)	Data yet to be reported.
C5 inhibition	Avacincaptad pegol	NCT03364153	Phase IIb (initiated 2017)	No data for STGD1 patients published.
	Eculzimab	No STGD trials	N/A	Well tolerated in AMD patients but no signs of efficacy.
Dietary				
	DHA	NCT00060749	Phase I (completed 2017)	Data for 11 STGD1 patients reported no adverse events or signs of efficacy [123]. Data for STGD3 cohort yet to be published.
	Omega-3 fatty acids	NCT03297515	Prospective trial completed 2021	Data yet to be reported.
	Saffron	NCT01278277	Phase I/II (initiated 2011)	No safety concerns and no significant changes in macular function of STGD1 patients after 6 months [125].
Cell replacement				
ESC-RPE	MA09-hRPE	NCT01625559	Phase I (initiated 2012)	No serious adverse events and no signs of efficacy 12 months post treatment in advanced STGD1 patients [128].
		NCT01345006	Phase I/II (completed 2021)	
		NCT01469832	Phase I/II (completed 2021)	Escalating dose of transplanted cells produced no serious adverse events with no signs of efficacy 12 months post treatment in 12 patients with advanced STGD1 [129].
		NCT02445612	Long-term follow-up to Phase I/II (initiated 2015)	Data yet to be reported.
	hESC-RPE	NCT02903576	Phase I/II (completed 2020)	Data yet to be reported.
		NCT02941991	Follow-up to Phase I/II (initiated 2016)	Data yet to be reported.
	BMSC	NCT01920867	Non-randomised open label (initiated 2013)	Data have been reported but issues exist regarding recruitment and study design [130].

**Table 3:** Summary of the different types of mutations that cause Stargardt disease. Data were extracted from ClinVar (June 2021) and only included mutations confirmed as pathogenic or likely path-ogenic with evidence of clinical phenotype. Numbers represent the different occurrences of each mutation type; for example, of 349 different *ABCA4* mutations, 72 were G > A transitions. ABE = adenine base editor, CBE = cytosine base editor.

Mutation	CRISPR Strategy	*ABCA4* Total = 349	*ELOVL4* Total = 23	*PROM1* Total = 3
G > A	ABE (coding strand) and RNA-ABE	72	3	1
A > G	CBE (non-coding strand)	21	1	0
T > C	CBE (coding strand)	31	3	1
C > T	ABE (non-coding strand)	63	4	1
G > T	Prime	20	2	0
G > C	Prime	14	0	0
T > A	Prime	12	0	0
A > T	Prime	8	1	0
C > A	Prime	15	2	0
A > C	Prime	4	1	0
C > G	Prime	13	4	0
T > G	Prime	17	0	0
Insert/deletion/duplication	Prime	59	2	0

## Data Availability

Not applicable. All data in this review are available from the sources stated in the manuscript.
